# Impact of *Ligilactobacillus salivarius* Li01 on benzo[*a*]pyrene-induced colitis, based on host-microbiome interactions in Mongolian gerbils

**DOI:** 10.3389/fnut.2025.1494525

**Published:** 2025-02-26

**Authors:** Yilun Huang, Can Yang, Bingmeng Fu, Honggang Guo, Yunxiang Chen, Dengfeng Xu

**Affiliations:** ^1^Alberta Institute, Wenzhou Medical University, Wenzhou, China; ^2^School of Laboratory Medicine and Bioengineering, Hangzhou Medical College, Hangzhou, China; ^3^Department of Nutrition, Child, and Adolescent Health, School of Public Health, Hangzhou Medical College, Hangzhou, China; ^4^Center of Laboratory Animal, Hangzhou Medical College, Hangzhou, China; ^5^Center for Safety Evaluation and Research, Hangzhou Medical College, Hangzhou, China

**Keywords:** *Ligilactobacillus salivarius* Li01, colitis, gut microbiota, SCFAs, probiotics

## Abstract

**Background:**

Probiotics supplementations have been regarded as an effective strategy for colitis treatment. However, the effect of *Ligilactobacillus salivarius* Li01 on benzo[*a*]pyrene (BaP)-induced colitis in Mongolian gerbils remains unclear. In this study, we leverage a BaP-induced model of colitis that exhibits significant remission following *Ligilactobacillus salivarius* Li01 intervention, to conduct an animal experiment that integrates histopathological assessment, inflammatory cytokines, 16S rRNA sequencing, targeted metabolomic profiling to investigate the relationship between *Ligilactobacillus salivarius* Li01, gut microbiota, and colitis.

**Results:**

We demonstrated that the improvements in colon histopathological assessment and inflammatory cytokines by *Ligilactobacillus salivarius* Li01 supplementation are accompanied by alterations in gut microbiota structure marked by increased abundance of strains with probiotic potential belonging to *Bifidobacterium* and *Eubacterium_coprostanoligenes*. Targeted metabolomic profiling analysis showed that *Ligilactobacillus salivarius* Li01 supplementation increases the concentration of acetic, propionic, butyric, and valeric acid. Correlation analysis showed that the alteration in the indicators associated with colitis is closely correlated to the changed microbial taxa and short-chain fatty acids (SCFAs).

**Conclusion:**

These data highlighted that *Ligilactobacillus salivarius* Li01 supplementation ameliorated the BaP-induced colitis, probably via modulating the structure of gut microbiota and promoting the production of SCFAs. Our findings provide preliminary evidence for a possible therapeutic strategy for the treatment of colitis based on host-microbiome interactions.

## 1 Introduction

Inflammatory bowel disease (IBD) is a group of chronic non-specific disorders affecting the ileum, cecum and colon, which includes Crohn’s disease (CD) and ulcerative colitis (UC). The pathogenesis of IBD is multifactorial and involves a complex interplay of gene mutations, eating habits, unbalanced gut microbiota, environmental hygiene and psychosocial factors (1). Over the last few decades, IBD has undergone considerable rises in Asia and other recently developed and developing countries since its emergence in the Western world (2). A 30-year cohort study from Korea indicated that the incidence of UC and CD patients was 6.58 and 2.42 in every 100,000 persons, respectively (3). In West Asia, the pooled annual incidence of UC was 2.33/100,000 persons and that of CD was 1.46/100,000 persons (4). In clinical practice, corticosteroids, immune suppressants and biological preparations are usually used for IBD treatment (5). However, these drugs are limited by their significant side effects during a long-time use. Therefore, it is important to find new strategies for managing IBD, which acts as a worldwide healthcare issue.

The gut microbiota is a community of more than 10^14^ bacteria residing in the intestinal tract (6). In recent years, impressive body of evidence indicating the crucial crosstalk between gut microbiota and IBD have emerged. For instance, Frank et al. (7) found a significant association between shifts in the relative abundance of *Faecalibacterium* and *Escherichia* taxa and the clinical IBD phenotype. In addition, previous studies have shown that IBD patients presented a distinct microbiota profile in which a reduction of Bacillota and Bacteroidota ratio, as well as some butyrate-producing genera such as *Eubacterium*, and an increase in Proteobacteria and Actinomycetota were observed (8). These dysbiosis may contribute to an elevation in intestinal barrier permeability, a symptom that is commonly observed in IBD. Evidence from epidemiological data has also indicated the crucial role of gut microbiota in the development of IBD (9). More importantly, a randomized controlled trial has revealed that receiving fecal microbiota transplantation (FMT) from a healthy donor can significantly decrease the endoscopic index of severity and the level of C reaction protein (CRP) in patients with IBD (10). All the existing data might be important because, in this content, new targets aimed at regulating the composition and metabolism of gut microbiota for IBD treatment could be furtherly explored.

Probiotics are defined as “living microorganisms” which can confer health benefits to the host when administered in sufficient amounts and duration (11). Studies have reported that some probiotics exhibit a variety of physiological functions that are beneficial to human health, such as antibacterial activity, anti-inflammatory activity, antioxidant activity and immunomodulatory activity (12, 13). Data from a meta-analysis of randomized controlled trials indicated that multi-strain probiotics supplementation can be regarded as a good choice in remission of IBD (14). Probably, the beneficial effects come from the fact that, probiotics can enhance the function of gut microbiota in improving intestinal barrier integrity and have a protective effect on systematic inflammation (15, 16). *Lactobacillus sali*var*ius* are probiotic strains widely used as a functional food and auxiliary medicine for various diseases (17). Current research has found that *Ligilactobacillus salivarius* Li01 could significantly alleviate the symptoms of acute liver injury in thioacetamide-induced mice by reducing serum inflammatory cytokine and lipopolysaccharide-binding protein (LBP) concentrations, and reshaping the perturbed gut microbiota (18). In addition, recent studies showed that *Ligilactobacillus salivarius* Li01 could significantly increase the ratio of Bacillota*/*Bacteroidota and improve the relative abundance of some beneficial genera such as *Rikenellaceae_RC9* and *Lachnospiraceae_NK4A136_group* (19, 20), suggesting the characteristics of probiotics. However, the effect of *Ligilactobacillus salivarius* Li01 on whether it could ameliorate gut inflammation and injury by regulating gut microbiota and its metabolites remains unclear in colitis models.

Mongolian gerbil (*Meriones unguiculatus*) is a kind of long-lived small mammal species, which can live up to 32 months in the wild, and up to 50 months in the laboratory, and are therefore used as biomedical research models for a variety of diseases. They are very susceptible to intestinal lesions induced by a chemical and sequential inflammation, and have beneficial applicability in a variety of tissue collection and intestinal pathology observations in comparison with common rodents (21). However, Mongolian gerbils are adapted to their original habitat, semi-deserts and steppes, and are able to reduce water uptake, which might interfere with the application of dextran sulfate sodium (DSS) via drinking water (22). Given this, we employed benzo[*a*]pyrene (BaP), a contaminant widely distributed in the human environment, to establish a colitis model (23). The present study aimed to investigate the effects of *Ligilactobacillus salivarius* Li01 supplementation on BaP-induced colitis in Mongolian gerbils and evaluate the role of gut microbiota. This study would therefore confirm the probiotic nature of *Ligilactobacillus salivarius* Li01 and provide new insight into the treatment of probiotics for colitis.

## 2 Methods

### 2.1 Probiotic strains and growth conditions

The probiotic strains used in present study were donated by Shaoxing Tongchuang Biotechnology Co., Ltd. (Shaoxin, China). Briefly, *Ligilactobacillus salivarius* Li01, originally isolated from the faeces of healthy individuals, was grown in Man-Rogosa-Sharpe (MRS) broth (OXOID, Hampshire, United Kingdom) medium in an anaerobic chamber (Electrotek Scientific, Shipley, United Kingdom) for 24 h at 37°C. The cultures were then centrifuged at 8,000 g for 10 min at 4°C. After that, the precipitate was washed twice with sterile phosphate-buffered saline (PBS) and resuspended to achieve a viable probiotic count of 5 × 10^10^ CFU/mL.

### 2.2 Animal experiments

**Figure 1:**
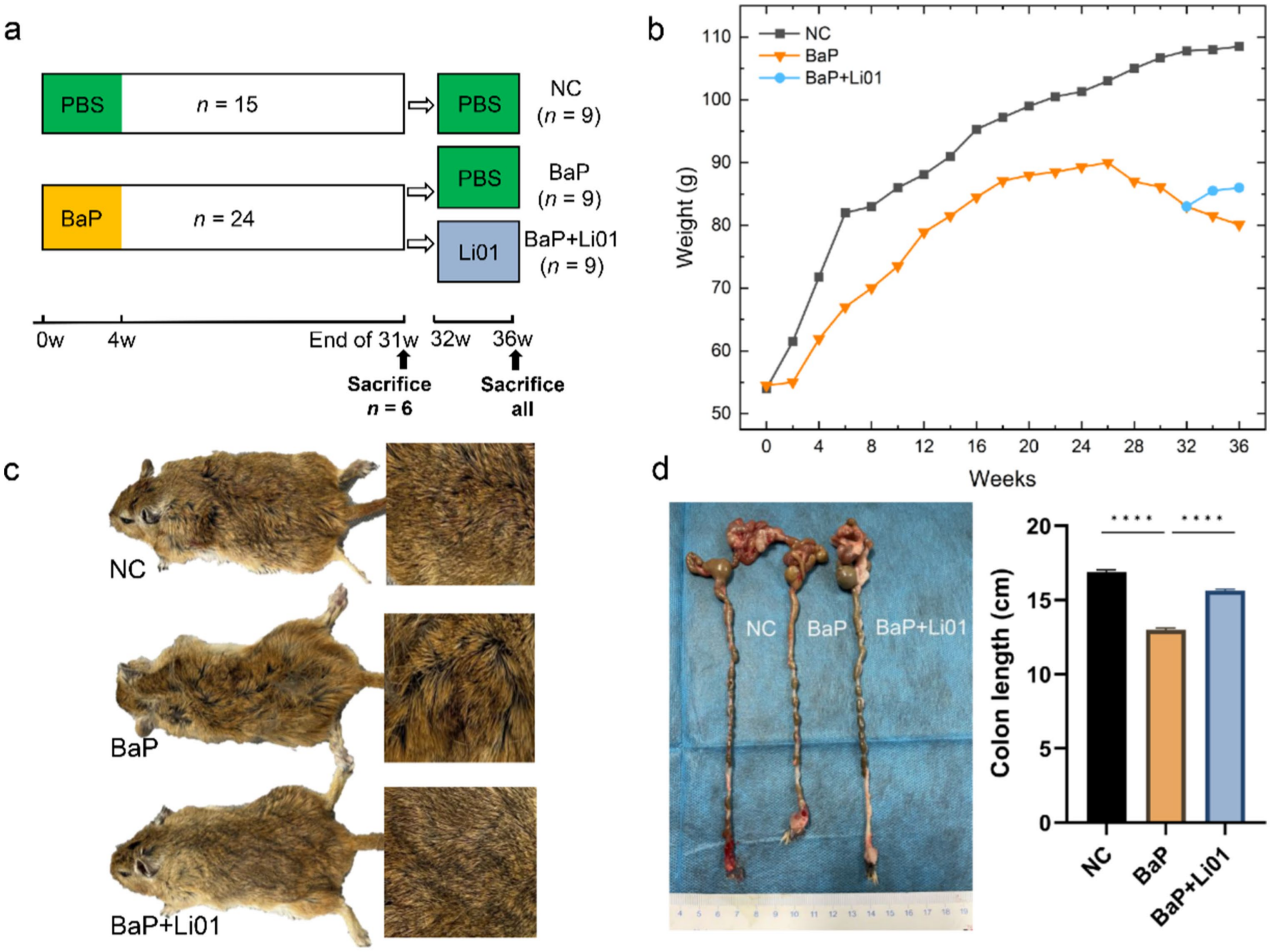
Effects of *Ligilactobacillus salivarius* Li01 on general physiology in Mongolian gerbils. **(a)** Study design of the animal experiment. **(b)** The body weight between groups during the whole experiment. **(c)** The general aspect between groups. **(d)** The colon length between groups. ^****^p < 0.0001.

Thirty-nine two-month-old male Mongolian gerbils were purchased from Zhejiang Provincial Laboratory Animal Center (License No. SCXK(ZHE)2019-0002) and housed in a conventional laboratory condition with a temperature of 22 ± 2°C, and a relative humidity of 50 ± 10%, with free access to water and food under a 12-h light/dark cycle. After 1 week of acclimatization, they were divided into two groups, normal control (NC, *n* = 15) group and model group (*n* = 24). During the first 4 weeks, to establish the colitis model, Mongolian gerbils in the NC group were orally administrated with 0.5 mL of PBS twice a week, while those in the model group were orally administrated with 0.5 mL of BaP (5 mg/mL, Sigma, B1760) twice a week (the initial body weight of each animal in the experiment was 50 ± 3 g) (23). Then at the end of week 31, six Mongolian gerbils from each group were sacrificed to verify whether the colitis model was successfully established. Finally, the remaining Mongolian gerbils in the model group were further divided into the BaP and the BaP + Li01 group, with the animals in the BaP group receiving 1 mL of PBS once a day and those in the BaP + Li01 group receiving 1 mL of *Ligilactobacillus salivarius* Li01 (5 × 10^10^ CFU/mL) once a day for 4 weeks. According to our previous studies, the beneficial effects of *Ligilactobacillus salivarius* Li01 are particularly significant at the current dosage. Furthermore, the amounts of probiotics currently consumed by the Chinese population also fall within this dosage range (19, 24). The body weight was measured once every 2 weeks. At the end of the whole experiment, all animals were euthanized, and all the indicators were compared. The animal experimental design is shown in Figure 1a. Animal protocols were approved by the Laboratory Animal Committee of Hangzhou Medical College (Hangzhou, China, approved number: 2023-115) and adhered to the guidelines of the Animal Ethical Committee.

### 2.3 Histopathological analysis

Hematoxylin-eosin (HE) staining was performed according to the manufacturer’s instruction. Briefly, the collected colons were dehydrated in graded alcohol (Macklin, E809056), then fixed with 4% paraformaldehyde (Biosharp, Beijing, China) and cut into 3 μm flakes. The slices were dewaxed with xylene, rewatered with ethanol and washed with distilled water. Thereafter, they were stained with hematoxylin for 5 min, and eosin for 2 min (Sigma-Aldrich). Finally, dehydration, tissue removal and sealing were carried out in order. The histopathological grading of the colon tissue was determined based on the guidelines outlined in a prior study (25). Briefly, three main categories sufficiently reflected the severity of histopathology independent of the localization and the overall extent of an inflammation: (i) quality and dimension of inflammatory cell infiltrates, (ii) epithelial changes and (iii) overall mucosal architecture. Scoring schemata were defined along specified criteria for each of the three categories. With a high degree of generalisation and maximum scores from 4–8 suitable scoring schemata accounted specific histopathological hallmarks. Besides, Mongolian gerbils were observed daily and scored for colitis severity using the disease activity index (DAI). The scores were based on weight loss, stool hardness, and presence of blood in the stool (13). Briefly, the scoring criteria were recorded as follows: (i): weight loss (0 score, zero; 1 score, 1–5% loss; 2 score, 6–10% loss; 3 score, 11–15% loss and 4 score more than 15% loss); (ii): stool consistency (0 score, normal; 1 score, slightly loose stool, 2 score, loose stools; 3 and 4 score, diarrhea); (iii): occult blood or gross bleeding (0 score, normal, 1 score, small presence of blood, 2 score, significant presence of blood; 3 and 4 score, gross blood). The colon length was measured without removing the fecal content by standardized procedures.

### 2.4 Enzyme-linked immunosorbent assay

Blood samples were collected from the orbital venous plexus of Mongolian gerbils and then centrifuged at 2,000 rpm for 15 min, and stored at −80°C until analysis. The levels of serum inflammation factors including TNF-α, IL-1β, IL-6, and IL-10 were measured via commercial kits (Proteintech. Co., Ltd. Rosemont, United States) according to the instructions of the manufacturer.

### 2.5 Detection of faecal short-chain fatty acid

Faeces samples (50 mg ± 10 mg) were extracted with 500 μL methanol (Thermo Fisher, United States)-water solution (containing 0.1% HCl, 20%H_2_O). After adding the acetic acid-D4 (CATO, China), the samples were crushed by a freezing crusher, ice bath Ultrasound 10 min, and then centrifuged (12,000 rpm, 4°C, 5 min). Take 200 μL of the upper liquid in the sample bottle, for GC-MS/MS analysis.

The GC–MS/MS consisted of a Trace 1,310 gas chromatography coupled to a TSQ 8000 Evo mass spectrometer (Thermo Scientific Technologies, United States). An Agilent Agilent HP-Innowax GC column (30 m, 0.25 mm ID, 0.25 μm film thickness) was employed with a sample injection volume of 1 μL and a splitless. Helium gas was used as carrier gas. The injection port temperature was set at 240°C. The column temperature was set to 50°C for 1 min and then increased to 180°C with a rate of 10°C/min, then increased to 240°C with a rate of 40°C/min, held at 240°C for 3 min. The MS spectra were acquired with the EI voltage of 70 eV.

External standard method was used for short-chain fatty acids (SCFAs) detection and is qualitatively compared to the retention time and SIM fragment ions by standard method. All mass spectrometry data acquisition and quantitative analysis of the target compound were performed with Thermo Scientific Xcalibur software.

### 2.6 Sequencing of 16S rRNA analysis

The bacterial 16S rRNA V3–V4 regions were amplified by using the primer V3_F341_N (5′-CCTACGGGNGGCWGCAG-3′) and V4_R805R (5′-GACTACHVGGGTATCTAATCC-3′). Sequencing was performed on the Illumina HiSeq platform by Hiseq 2500. The Hiseq analysis was based on nine parallel samples for each group. Sequences with a similarity of more than 97% could be classified as the same operation taxonomic units (OTUs). α-diversity, which reflects the diversity (Shannon or Simpson index) of microbial communities that existed in a single sample, could be calculated by Mothur (26). Furthermore, we employed multiple response permutation procedures, and principal coordinate analysis (PCoA) to calculate β-diversity, which indicated the similarity of diversity between groups.

### 2.7 Statistical analysis

Data are shown as mean ± SEM if it follows a normal distribution, otherwise, median and interquartile range was used. One-way ANOVA or the Kruskal–Wallis test was utilized to test significant differences among multiple groups and post-multiple comparison (between NC and BaP group, and between BaP and BaP + Li01 group) was assessed by Dunnett’s *t*-test. The linear discriminant analysis (LDA) effect size (LEfSe) was performed to identify the significantly abundant taxa of bacteria among the three groups (LDA score > 4.0, *p* < 0.05). Spearman’s correlation analysis was conducted to evaluate the relationship between key gut microbiota and colitis indicators. All analyses were performed by using GraphPad Prism 9.5.1 (GraphPad Software Inc., San Diego, CA, United States), Origin64, and R software. *p* < 0.05 was considered statistically significant.

## 3 Results

### 3.1 Effects of *Ligilactobacillus salivarius* Li01 on general physiology in Mongolian gerbils

As shown in Figure 1c, compared with the NC group, Mongolian gerbils in the BaP group exhibited signs of compromised health, characterized by disheveled fur, diarrhea, diminished activity, and a general state of lethargy. After *Ligilactobacillus salivarius* Li01 intervention, significant improvements with alleviated diarrhea and improved behavioral activity were observed. In terms of body weight, *Ligilactobacillus salivarius* Li01 can prevent the weight loss induced by BaP administration and exhibit a gradual weight gain (Figure 1b). In addition, the decreased colon length induced by BaP was significantly reversed by *Ligilactobacillus salivarius* Li01 at the end of the interventional phase (*p* < 0.0001, Figure 1d). These findings indicated a preliminary beneficial impact of the probiotic on host health.

### 3.2 Effects of *Ligilactobacillus salivarius* Li01 on BaP-induced histopathological assessment in colon

**Figure 2:**
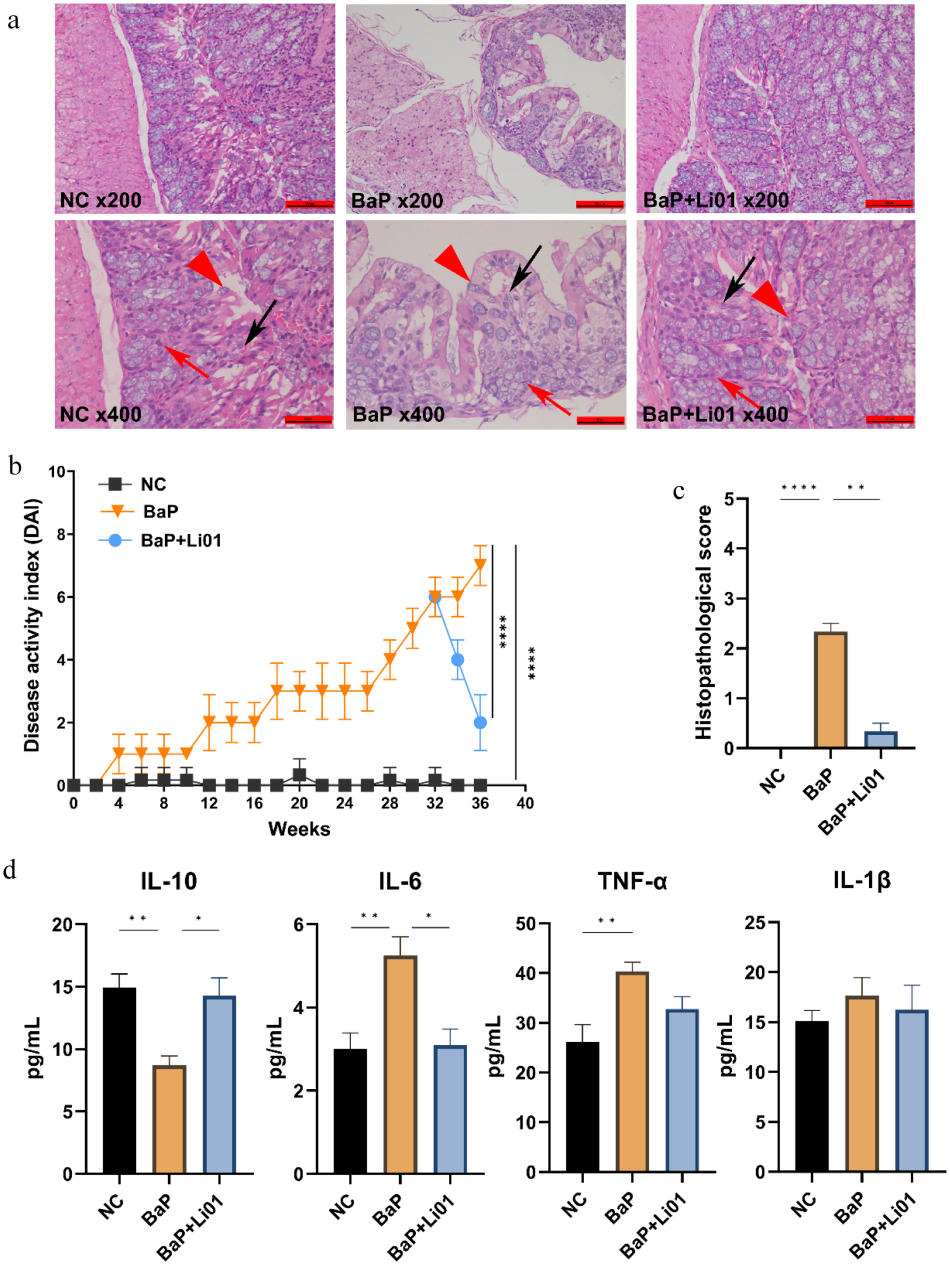
Effects of *Ligilactobacillus salivarius* Li01 on histopathological assessment and inflammatory cytokines in Mongolian gerbils. **(a)** Histopathological assessment between groups. **(b)** The DAI values between groups. **(c)** The histopathological score between groups. **(d)** The inflammatory cytokines between groups. ^**^p < 0.01 and ^****^p < 0.0001.

To determine histological changes in the colon, HE staining was conducted. As shown in Figure 2a, the NC group had a regular histological architecture, characterized by intact epithelium and crypts, and abundant goblet cells. In contrast, the BaP group presented significant inflammation and cellular infiltration, deteriorating crypts and goblet cells, and disrupted epithelium tissue. On the other hand, in comparison with the BaP group, *Ligilactobacillus salivarius* Li01 intervention led to less damage on colonic tissue; moreover, mild inflammatory infiltration, and more intact crypt structures were also observed. These findings were furtherly proved by the results from the pathological score of colon tissue, in which the BaP group had the highest scores and markedly reduced grade in the BaP + Li01 group (*p* = 0.0012, Figure 2c). Similarly, compared with the BaP group, the BaP + Li01 group had significantly reduced DAI values (*p* < 0.001, Figure 2b). All above, the results showed that the supplementation of *Ligilactobacillus salivarius* Li01 had a protective effect on BaP-induced colitis.

### 3.3 Effects of *Ligilactobacillus salivarius* Li01 on inflammatory cytokines

Figure 2d presented the results for the effects of *Ligilactobacillus salivarius* Li01 on systematic inflammation in Mongolian gerbils. A notable decrease in the level of IL-6 was found in the BaP + Li01 group compared with the BaP group (*p* = 0.0026). Besides, compared to the BaP group, the anti-inflammatory factor, IL-10 for the BaP + Li01 group displayed a significant increase (*p* = 0.0031). However, there were no significant differences in TNF-α and IL-1β between BaP and BaP + Li01 group (*p* = 0.1353 and *p* = 0.8556, respectively). These results exhibited the crucial role of *Ligilactobacillus salivarius* Li01 in ameliorating BaP-related inflammation.

### 3.4 Effects of *Ligilactobacillus salivarius* Li01 on gut microbiota

**Figure 3:**
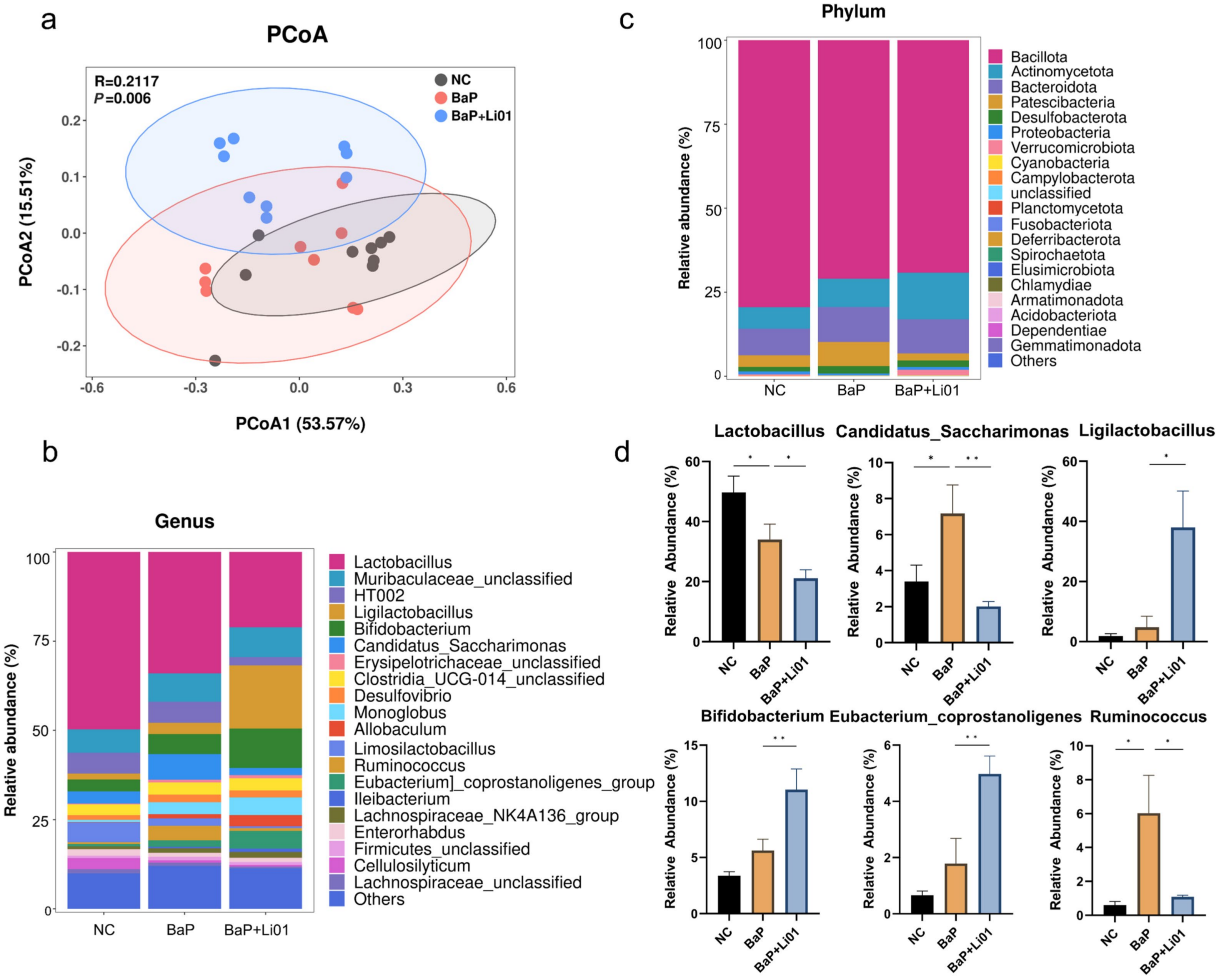
Effects of *Ligilactobacillus salivarius* Li01 on gut microbiota in Mongolian gerbils. **(a)** PCoA analysis. The relative abundance of top 20 phyla in each group at genus **(b)** and phylum **(c)** level. **(d)** The significant abundant gut microbiota between groups. ^*^p < 0.05 and ^**^p < 0.01.

To investigate the change in gut microbiota after *Ligilactobacillus salivarius* Li01 supplementation, we conducted a pyrosequencing-based analysis of bacterial 16S rRNA by using Illumina Hiseq to identify the modulated effect of *Ligilactobacillus salivarius* Li01 on intestinal bacteria. The violin diagram showed that the averaged numbers of OTUs in the NC, BaP and BaP + Li01 group were 461, 462 and 508 (Supplementary Figure 1). In terms of α-diversity, a total of 3 indexes (Shannon, Simpson, and Chao1) were employed to assess the species richness. The results showed that no significant difference was observed between BaP and BaP + Li01 group (Supplementary Table 1). Next, we performed PCoA analysis based on weighted UniFrac distance to explore changes in the microbiota structure. As shown in Figure 3a, the model suggested that the BaP + Li01 group had a significant separation trend from the BaP group, among which the first and second principal coordinate in the PCoA analysis could explain 53.57% and 15.51% of the overall variations, respectively. These results provided evidence for the modulating effect of *Ligilactobacillus salivarius* Li01 on microbial β-diversity.

The histogram illustrating the species and relative abundance of gut microbiota at the genus and phylum level is given in Figures 3b,c. Briefly, *Lactobacillus* and Bacillota were the dominant species at genus and phylum, respectively. Significant reductions in the *Lactobacillus*, *Ruminococcus* and *Candidatus Saccharimonas* were observed in BaP + Li01 compared with the BaP group. In addition, it seems that *Ligilactobacillus salivarius* Li01 could down-regulate *Ruminococcus* to levels more similar to those of the NC group. In contrast, the relative abundance of both *Ligilactobacillus* and *Bifidobacterium* had significant increases after *Ligilactobacillus salivarius* Li01 treatment (Figure 3d). However, no significant between-group differences in *Desulfovibrio* and other bacterial genera were found after the intervention (data not shown).

**Figure 4:**
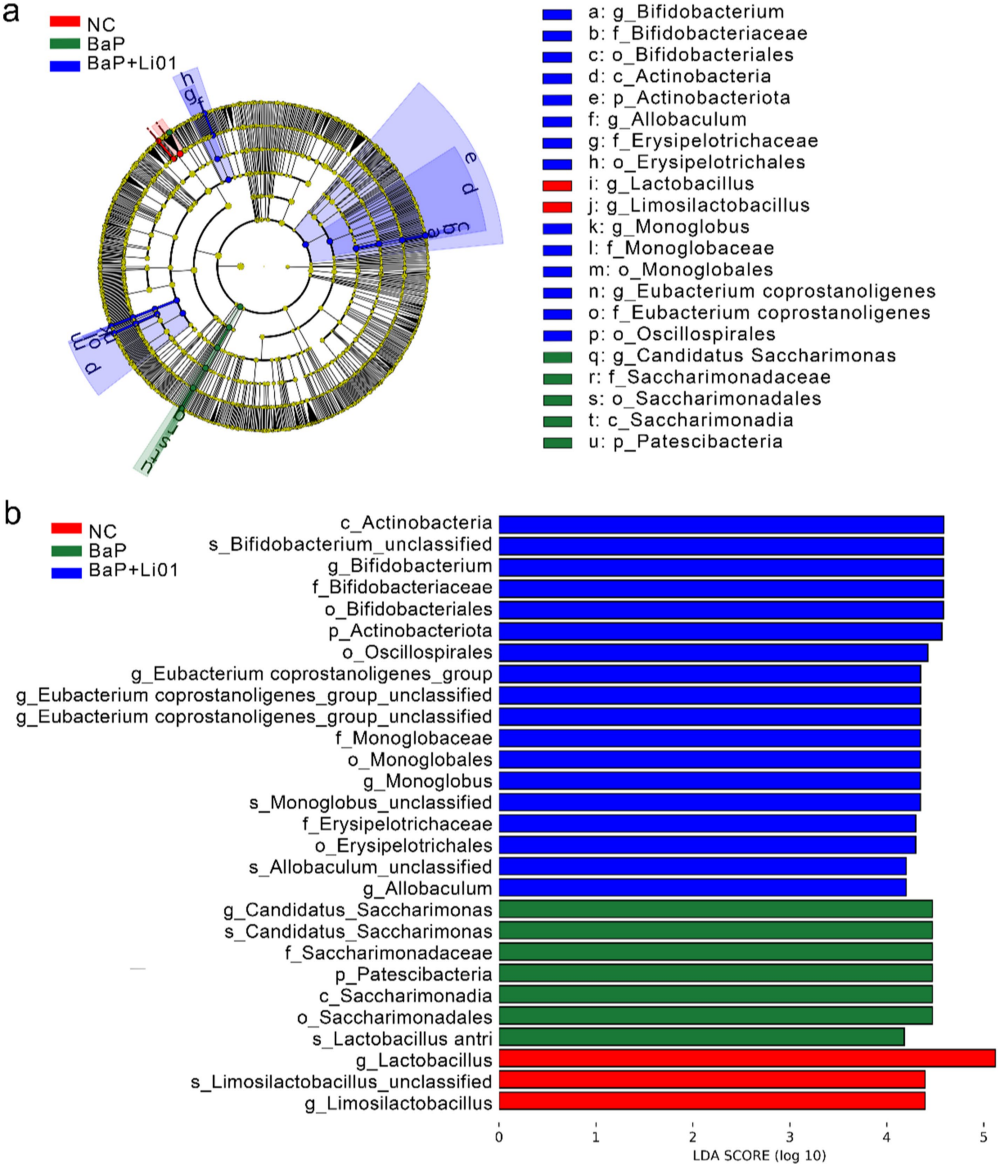
The results of a LEfSe analysis through **(a)** cladrogram and **(b)** LDA score of *Ligilactobacillus salivarius* Li01 on gut microbiota in Mongolian gerbils.

Figure 4 showed the results of the LEfSe and LDA analyses. The relative abundance of *Lactobacillus* was higher in NC group. However, the BaP group showed increased abundances of potentially pathogenic bacteria, such as *Candidatus Saccharimonas* at genus level, and Saccharimondia at class level. Increased abundances of potentially beneficial bacteria were observed in the BaP + Li01 group, such as *Bifidobacterium*, and *Eubacterium_coprostanoligenes*. In brief, intervention with *Ligilactobacillus salivarius* Li01, leads to significant changes in gut microbiota and could largely reverse the gut dysbiosis associated with colitis.

### 3.5 Effects of *Ligilactobacillus salivarius* Li01 on faecal SCFAs

**Figure 5:**
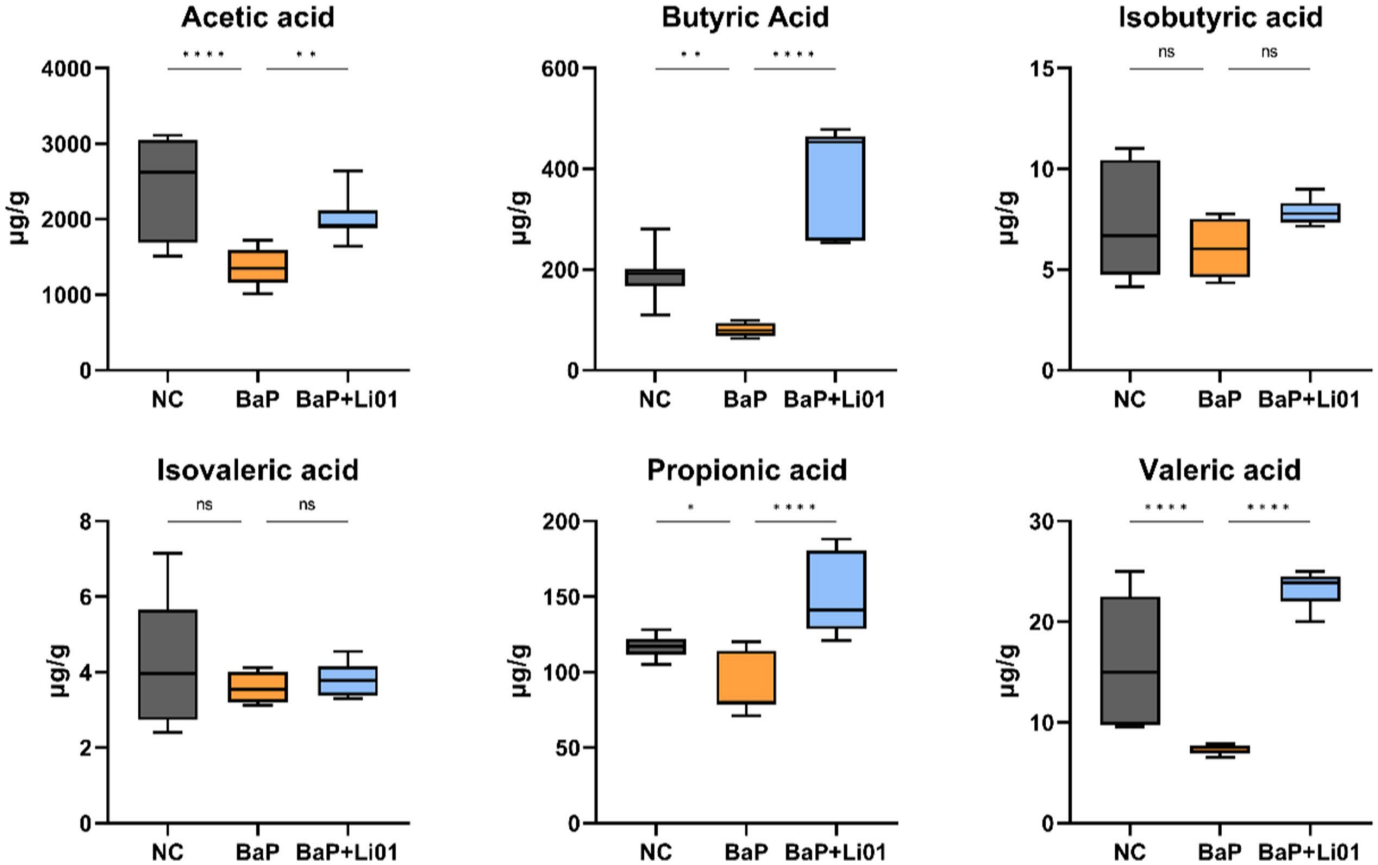
Effects of *Ligilactobacillus salivarius* Li01 on SCFAs in Mongolian gerbils. ^*^p < 0.05, ^**^p < 0.01, ^***^p < 0.001, and ^****^p < 0.0001.

Since SCFAs play a crucial role in maintaining gut health, we measured the concentration of SCFAs in faeces. As shown in Figure 5, the levels of acetic, propionic, butyric and valeric acid were significantly increased in the BaP + Li01 group compared to the BaP group. While the levels of isobutyric and isovaleric acid were not significantly changed between groups.

### 3.6 Correlation analysis between gut microbiota, SCFAs, histopathological indicators, and inflammatory cytokines

**Figure 6:**
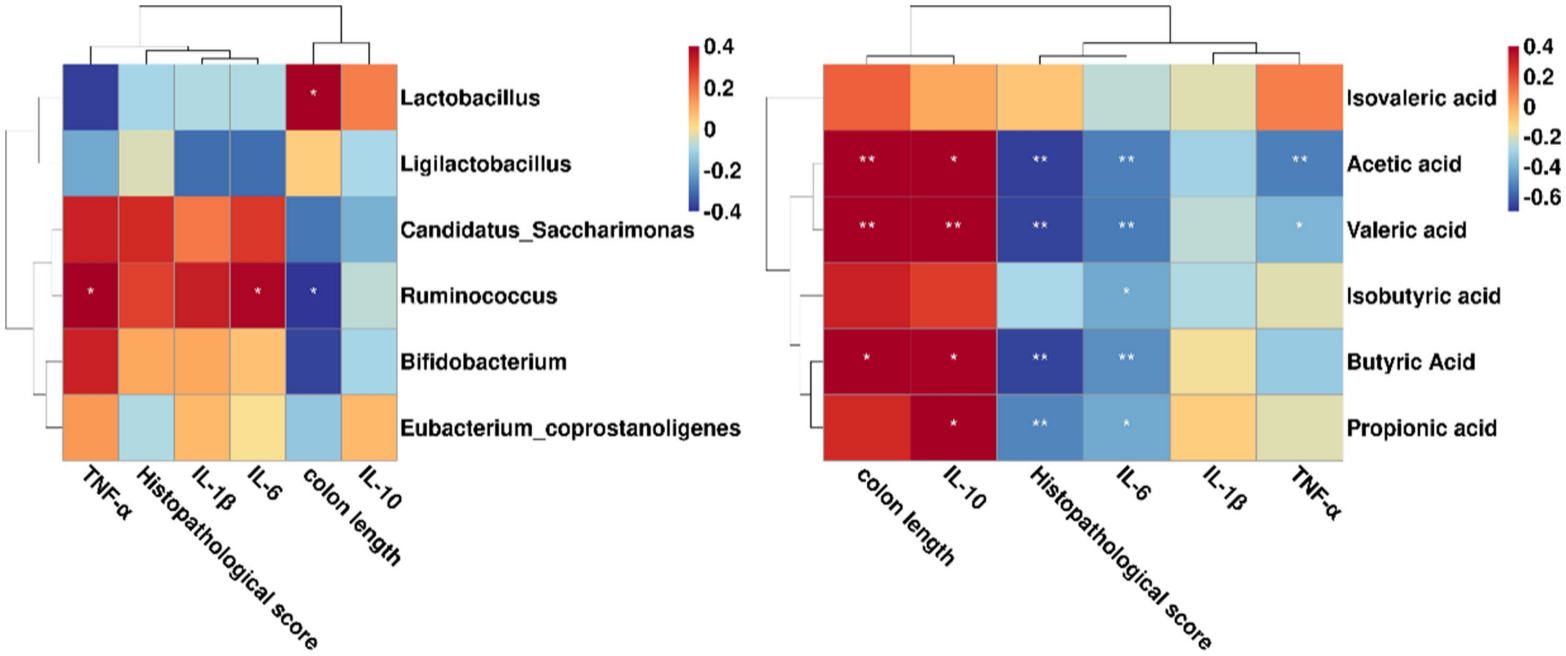
Correlations between the key microbiota, SCFAs and colitis indicators in Mongolian gerbils. ^*^p < 0.05 and ^**^p < 0.01.

Furtherly, we employed the Spearman’s correlation analysis to investigate the relationships between the key microbiota, SCFAs and colitis indicators. As shown in Figure 6, *Ruminococcus* were notably in negative and positive correlation with colon length and IL-6, respectively. Of note, *Lactobacillus* was positively associated with colon length. However, when we focused only on BaP + Li01 group, we found that the relative abundance of *Bifidobacterium* was negatively correlated with TNF-α, while *Ligilactobacillus* was negatively associated with IL-6 (data not shown). On the other hand, we found that acetic acid was negatively associated with IL-6 and histopathological score, while positively correlated with colon length and IL-10. Additionally, there were obvious negative correlations between propionic acid and histopathological score, as well as IL-6, while positive correlation with IL-10. Similar results were also observed in the relationship between butyric acid and colitis indicators (Figure 6). These results suggested that some gut microbiota could reduce gut inflammation via producing SCFAs and improving intestinal pathology.

## 4 Discussion

Besides host genetics and environmental factors, in recent years, the role of probiotics supplementation in UC has gained popularity due to its beneficial health properties on gut microbiota. Many studies have demonstrated that probiotics supplementation is an effective intervention for IBD patients (27, 28). Our study aimed to evaluate the alleviating effect of *Ligilactobacillus salivarius* Li01 in Mongolian gerbils by using a BaP-induced colitis model. The results suggested that oral administration of BaP to Mongolian gerbils for 4 weeks destroyed the intestinal epithelial tissue and led to inflammation. We also found that oral administration of *Ligilactobacillus salivarius* Li01 could significantly prevent weight loss and improve the symptoms of colitis characterized by decreased inflammatory infiltration, improved crypt structures and restored number of goblet cells. In addition, 16S rRNA sequencing and Spearman’s correlation analysis furtherly suggested the beneficial effects of *Ligilactobacillus salivarius* Li01 might be related to the modulated gut microbial community and its metabolites of SCFAs.

Body weight is a reliable indicator reflecting the severity of colitis in animals. In present study, we found that the Mongolian gerbils in the BaP group lost more than 20% of their body weight compared to the NC group. While the Mongolian gerbils with *Ligilactobacillus salivarius* Li01 supplementation for 4 weeks gradually regained their body weight. These results were in accordance with Shi et al. (24) who reported that *Ligilactobacillus salivarius* Li01 had significant capacity to protect against the weight loss induced by CCl_4_, indicating a preliminary therapeutic effect for colitis. Meanwhile, the increased colon length and lower scores in pathological evaluation in the BaP + Li01 group furtherly supported the beneficial effect.

Previous studies have indicated that inflammation cytokines play a crucial role in the pathogenesis of colitis (29, 30). We did find that, compared with the NC group, the levels of pro-inflammatory cytokines such as IL-6 and TNF-α were significantly increased in the BaP group. After a 4-week *Ligilactobacillus salivarius* Li01 intervention, the concentration of IL-6 showed a significant decrease in BaP + Li01 group, while IL-10 was significantly increased. These results showed a high consistency with a meta-analysis that had confirmed the efficacy of probiotic supplementation in ameliorating systematic inflammation (31). Just as found by Yan et al. (32), who reported that the protein p40 secreted by *Lactobacillus rhamnosus GG* can activate the epidermal growth factor receptor (EGFR) pathway, thereby enhancing host immune function and protecting against experimental colitis. Despite this, we failed to find a statistical significance in the level of TNF-α and IL-1β between the BaP and BaP + Li01 group. With this in mind, our study highlights the need for further mechanistic researches.

Actually, gut microbiota has been regarded as a key factor in the development of colitis (33, 34). A two-sample Mendelian randomization study has suggested a potential causal association between gut microbial genera and UC in humans (35). In the present study, we observed significant between-group differences in microbial composition using PCoA analysis, which indicates the regulated effect of *Ligilactobacillus sali*var*ius* Li01 on microbial β-diversity. This result is consistent with Qiu’s et al. (20) findings, in which *Ligilactobacillus salivarius* Li01 also exhibited a significant difference in microbial composition in Li01 group compared to other group. However, no significant difference in α-diversity was observed between BaP and BaP + Li01 groups. We believed it may happen since minor changes in distinct taxonomic groups could be masked by high interindividual variations among the animals.

In our BaP-induced colitis in Mongolian gerbils, the abundance of several genera, such as *Candidatus Saccharimonas* and *Ruminococcus* increased significantly, and then decreased by *Ligilactobacillus salivarius* Li01 treatment. According to a previous study, *Candidatus Saccharimonas* is reported to be closely related to inflammatory mucosal diseases and may be a key component of colitis-associated carcinogenesis (36). Mechanistic study demonstrated that *Candidatus Saccharimonas* could suppress the production of TNF in macrophages, indicating a potential capacity for immune suppression (37). In other words, *Candidatus Saccharimonas* may have its growth favored by the presence of an inflammatory condition. On the other hand, we found that supplementation with *Ligilactobacillus salivarius* Li01 prevented BaP-induced intestinal inflammatory disorder, as assessed by a decreased level of IL-6 and a reduced abundance of *Candidatus Saccharimonas*. We hypothesize that the phenomenon might be related to the anti-inflammatory property of *Ligilactobacillus salivarius* groups (38, 39). The abundance of *Ruminococcus*, which can exhibit pro-inflammation response, has been shown to increase significantly in patients with IBD (40). Furthermore, correlation analysis indicated that *Ruminococcus* was positively correlated with IL-6, and inversely correlated with colon length. These findings were consistent with Li et al. (41), in which the authors found that the levels of IL-6 in the osteoporosis model in mice were significantly upregulated with a higher abundance of *Ruminococcus*. Interestingly, we observed a notable reduction in the abundance of *Lactobacillus* in the BaP + Li01 group, which conflicts with the others to some extent (42). Given that the probiotics used in the current study are one of the subtypes of *Lactobacillus*, probably, supplementation of *Ligilactobacillus salivarius* Li01 might mask the relative abundance of *Lactobacillus*. This hypothesis was partly supported by the increased abundance of *Ligilactobacillus* in the BaP + Li01 group. Furthermore, due to the significant shifts in gut microbiota composition, the relationship among certain altered genera may become complicated. For instance, the increased abundance of *Eubacterium_coprostanoligenes* observed in BaP + Li01 group may disrupt *Lactobacillus* by competing for nutrients, given that both species possess the ability to ferment carbohydrates. This hypothesis has received partial support from the most recent studies as well (43, 44). In further study, we aimed to explain the exact association between changes in the specific gut microbiota and probiotics supplementation.

In fact, the mechanisms through which gut microbiota affects colitis are not well understood. One possible pathway is SCFAs, which are produced by bacterial fermentation of indigestible carbohydrates (45). Many studies have shown that SCFAs are involved in the development and aggravation of colonic inflammation (46). It is reported that SCFAs are potentially beneficial for gut barrier integrity by stimulating the production of mucus and tightening the junctions between intestinal cells (47). In our study, we found a significant increase in faecal level of SCFAs mainly including acetic, propionic and butyric acid after *Ligilactobacillus salivarius* Li01 intervention. Furthermore, the level of main SCFAs showed significant inverse associations with IL-6 and histopathological score, and positive correlation with colon length and IL-10, indicating a good anti-inflammatory characteristic. In a similar study, *Saccharomyces cerevisiae* I4 treatment significantly increased faecal SCFA levels and greatly reduced inflammatory cytokines, compared with the model group drinking 3% DSS water without yeast treatment (48). Indeed, in a mice experiment of DSS-induced colitis, Tong et al. (49) found that supplementation of propionate acid could ameliorate dextran DSS-induced colitis by reducing inflammation and improving intestinal barrier function through the STAT3 signaling pathway. We speculated that the increased levels of SCFAs might be a consequence of the increased relative abundance of a group of SCFA-producing bacteria, such as *Bifidobacterium*. In comparison with the BaP group, a significant increase of *Bifidobacterium* was observed in the BaP + Li01 group. Moreover, regarding the composition of gut microbiota, we found that the relative abundance of *Ligilactobacillus*, and *Eubacterium_coprostanoligenes* were also increased with *Ligilactobacillus salivarius* Li01 supplementation although the correlation analysis did not suggest some significant findings. These genera are believed to have the capacity to produce SCFAs and maintain gut health (50, 51). In contrast, we found a notable positive correlation between *Lactobacillus* and colon length despite the fact that there was a significant reduction in the relative abundance of *Lactobacillus* in the BaP + Li01 group. These results conflict with previous studies to some extent, since *Lactobacillus* was proved to have properties of regulating the intestinal pH and relieving inflammation (52). In summary, all these findings supported the crucial role of SCFAs stimulated by gut microbiota in ameliorating indicators associated with colitis.

There are some limitations that should be noted. One limitation of this study was that we did not examine the expressions of gut barrier-related proteins such as claudin-1 and occludin. A well experimental design would be necessary in the future. In addition, we could not conclude that there is a causal relationship between gut microbiota and improvement in colitis indicators. Further FMT experiment is necessary to clarify whether the beneficial effects rely on the modulated gut microbiota. Finally, given that the current study primarily emphasizes the therapeutic effects of *Ligilactobacillus salivarius* Li01, its potential early preventive role in colitis remains largely unknown. Consequently, it is required to conduct research involving the role of *Ligilactobacillus salivarius* Li01 in the initial stages of colitis.

## 5 Conclusion

In conclusion, we demonstrated that *Ligilactobacillus salivarius* Li01 supplementation exerts protective effects against BaP-induced colitis in Mongolian gerbils. *Ligilactobacillus salivarius* Li01 alleviated intestinal damage and intestinal inflammation. Meanwhile, *Ligilactobacillus salivarius* Li01 restored the dysbiosis of gut microbiota and improved the concentration of SCFAs. Correlation analysis suggested that these effects might involve with the regulated gut microbial community and its metabolites SCFAs, which provides a new strategy for IBD treatment and extends our understanding of the role of gut microbiota in ameliorating BaP-induced colitis.

## Data Availability

The datasets presented in this study can be found in online repositories. The names of the repository/repositories and accession number(s) can be found in the article/Supplementary material.

## Supplementary material

SUPPLEMENTARY FIGURE 1 The numbers of OTUs in each group.

SUPPLEMENTARY TABLE 1 The indexes of α-diversity in each group.
