# Supplementary material for: Impact of Ligilactobacillus salivarius Li01 on benzo[a]pyrene-induced colitis, based on host-microbiome interactions in Mongolian gerbils
Source: Front Nutr. 2025 Feb 26;12:1494525. doi: 10.3389/fnut.2025.1494525 (PMC11896860; doi:10.3389/fnut.2025.1494525)
Supplement: Supplementary file 1 [file Data_Sheet_1.docx]

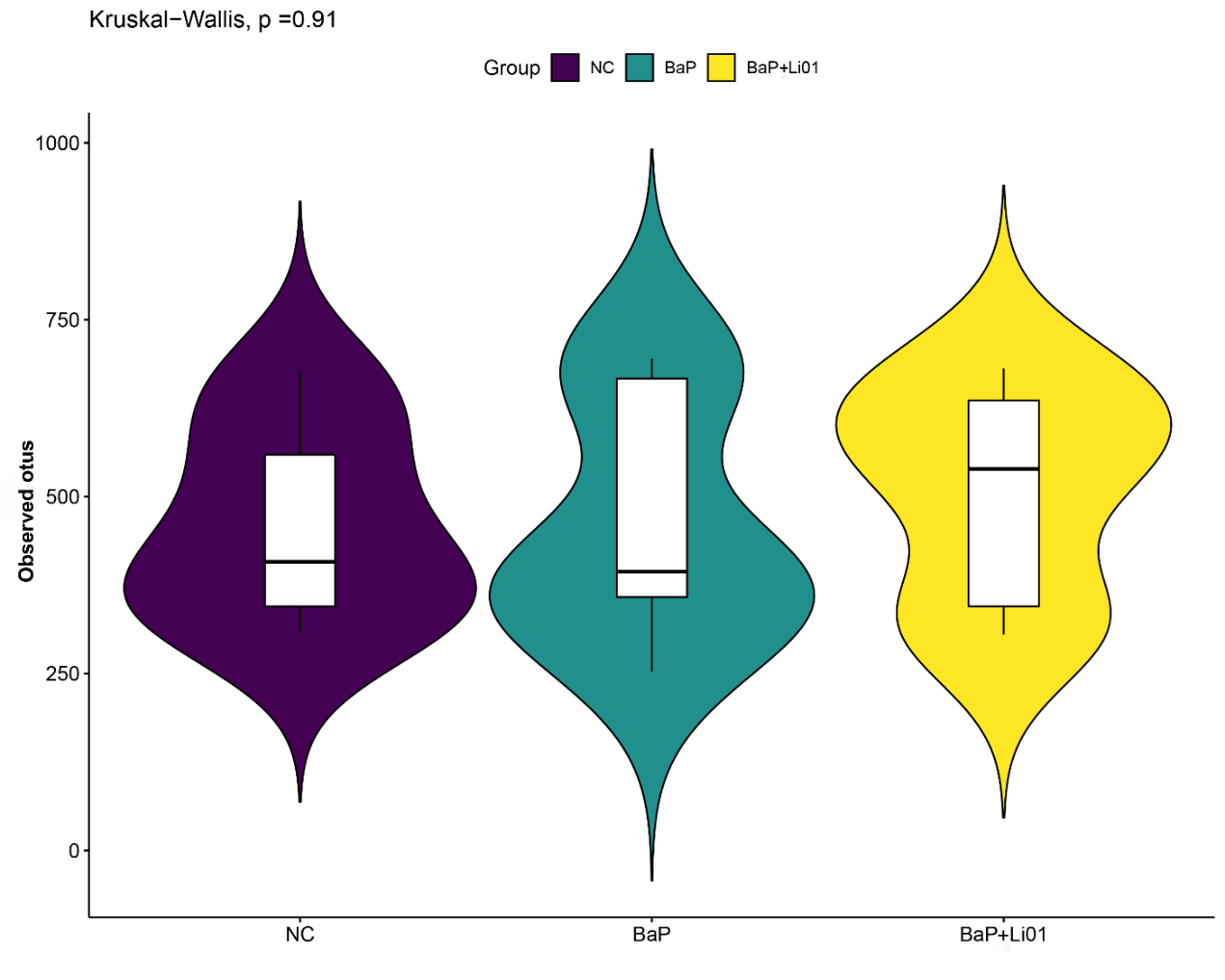
Supplementary Figure 1 The numbers of OTUs in each group

Supplementary Table 1 The indexes of α-diversity in each group

| **Groups** | **Shannon** | **Simpson** | **Chao1** |
| --- | --- | --- | --- |
| **NC** | **4.398±1.077** | **0.794±0.084** | **462.297±137.762** |
| **BaP** | **5.386±1.042** | **0.909±0.063*** | **463.256±170.399** |
| **BaP+Li01** | **5.539±1.167** | **0.901±0.081** | **509.256±148.994** |

Values shown are mean ± SD, *n* = 9.

^⁎^ *p* < 0.05, as compared with the NC group.
